# Using the promoters of MerR family proteins as “rheostats” to engineer whole-cell heavy metal biosensors with adjustable sensitivity

**DOI:** 10.1186/s13036-019-0202-3

**Published:** 2019-08-20

**Authors:** Mingzhang Guo, Ruoxi Du, Zixin Xie, Xiaoyun He, Kunlun Huang, Yunbo Luo, Wentao Xu

**Affiliations:** 1 Beijing Advanced Innovation Center for Food Nutrition and Human Health, College of Food Science and Nutritional Engineering, China Agricultural University, Beijing, 100083 China; 2 Key Laboratory of Safety Assessment of Genetically Modified Organism (Food Safety), Ministry of Agriculture, Beijing, 100083 China

**Keywords:** Whole-cell biosensor, Circuit design principle, Heavy metals, MerR family

## Abstract

**Background:**

Whole cell biosensors provide a simple method for the detection of heavy metals. However, previous designs of them rely primarily on simulation of heavy metal resistance systems of bacteria.

**Results:**

This study proposes a strategy for the rational design of metal detection circuits based on sensor proteins of the MerR family. Our results indicate the expression level of sensor protein can be used as a “rheostat” for tuning detection sensitivity with parabola curves to represent the relationships between the detection slopes and the sensor protein levels. This circuits design strategy (named as “Parabola Principle”), is used as a guide for the discovery of optimum metal detection circuits, and the design of biosensors with specific metal detection characteristics. For example, visible qualitative Hg (II) biosensors with a threshold of 0.05 mg/L are successfully constructed.

**Conclusions:**

These results indicate the feasibility of developing a sensor that is much more tunable than what is presented.

**Graphical abstract:**

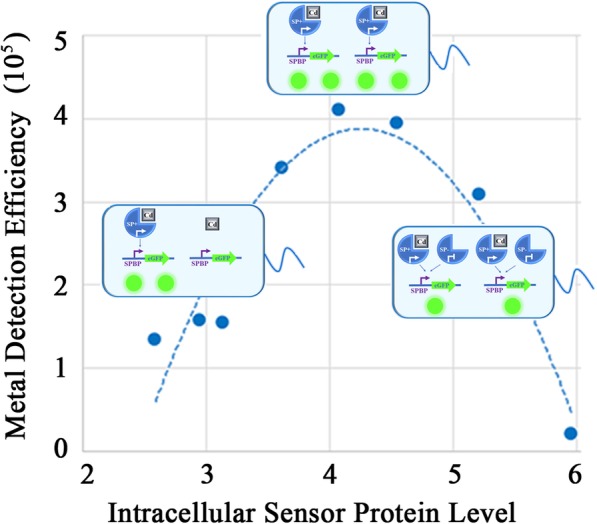

**Electronic supplementary material:**

The online version of this article (10.1186/s13036-019-0202-3) contains supplementary material, which is available to authorized users.

## Background

Heavy metal pollution in water and soil has showed a steady increase in recent years due to growing anthropogenic activities. Furthermore, the abundance of these pollutants in grains and animals utilized for consumption, poses a severe threat to human health and the social economy [1]. Therefore, the development of rapid, simple, and cost-effective detection methods will promote the management and remission of heavy metal pollution. Whole-cell metal biosensors provide a cheap and uncomplicated method for the detection of heavy metals in a variety of samples [2]. Most whole-cell metal biosensors simulate the heavy metal resistance systems of bacteria. These mechanisms include sensor proteins (SPs) to discern the intracellular metal concentration, downstream effector proteins (EPs) to reduce the toxicity levels of heavy metals in bacterial cells, as well as SPs binding promoters (SPBP) that are regulated by SPs and initiate the expression of EPs. By replacing the EPs with reporter proteins such as fluorescent proteins and luciferases, these microbial, heavy metal resistance systems can be reconstructed as heavy metal detection circuits, such as biosensors for the recognition of cadmium [3–6], mercury [7–9], lead [10–12], and zinc [13, 14].

Although whole-cell metal biosensors show considerable promise for on-site utilization, many areas require improvement to achieve successful practical application. An undeniable challenge is the lack of systematic theories providing guidelines regarding the rational design process of metal detection circuits. For example, several earlier metal detection circuits employed native promoters to initiate the expression of SPs [5, 11, 12, 15]. However, these native promoters evolved to be optimal for bacterial survival, and may no longer be suitable for utilization in metal detection biosensors. Furthermore, since the recommended maximum exposure of different samples vary considerably [1], the application of biosensors with diverse linearities are necessary, while an approach regarding the rational design of biosensors with a specific detection range and sensitivity remain unclear.

SPs are core elements of whole-cell metal biosensor circuits. The MerR family is a collection of SPs present in bacteria including *cadR*, *merR*, *cueR*, *pbrR*, *zntR*, *hmrR*, *pmtR, nimR* and more [16]. These SPs bind to the spacing region between the − 35 and − 10 elements of the corresponding SPBP. They either act as strong activators in the presence of metal ions or as slight repressors in the absence of metal ions [17–19]. The MerR family presents a vital component source in designing metal biosensors [16]. In this study, a series of constitutive promoters were used to express a variety of SPs belonging to the MerR family. This process was employed to analyze the relationship between the intracellular levels of the SPs and the linear characteristics of heavy metal biosensors. Our results showed that the expression level of sensor protein can be used as a regulator in the genetic circuits for tuning detection sensitivity, similar to a “rheostat” which is used to control current in the electrical circuits.

## Results and discussion

### Construction of plasmids and Design of Promoters

**Fig. 1:**
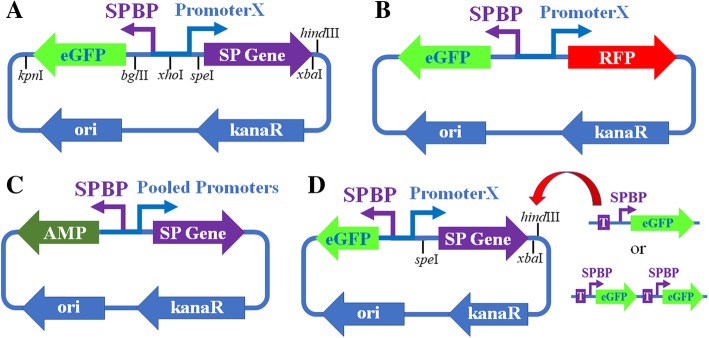
Plasmids constructed in this study. (**a**) pMetalBasic, (**b**) pMetalRFP, (**c**) pMetalAMP, (**d**) pMetalMultiGFP. SP: Sensor Protein, SPBP: Sensor Protein Bind Promoter, kanaR: Kanamycin resistance gene, AMP: ampicillin resistance gene

Plasmid pMetalBasic (Fig. 1a) was constructed as the basic plasmid, in which Promoter X was used to initiate the expression of SP genes, while SPBP was used to initiate the expression of eGFP. All plasmids used in this study were constructed by replacing the circuit elements of pMetalBasic. For the Cd (II), Pb (II), and Cu (I) biosensor frame, *cadR*, *pbrR*, and *cueR* were individually used as SP genes, and their respective binding promoters were employed as SPBPs (Additional file 2: Table S1-S2).

This research employed a thermodynamic model to design a series of constitutive promoters according to Brewster’s report [20]. To confirm the accuracy of this model within the plasmid system of this particular study, seven promoters were designed by Brewster et al. and referred to as Constitutive Promoter Subseries I in this article (Additional file 2: Table S3), with promoter names indicating the model predicted expression levels × 100, were used to express RFP in the plasmid pMetalRFP (Fig. 1b). The log values of RFP expression levels (calculated by Log(RFP fluorescence intensities) + 4.5) by these promoters showed linear correlation with the expression levels predicted by this model (R^2^ = 0.9077, Additional file 1: Figure S1A), as well as with the LacZ expression levels reported by Brewster et al. (R^2^ = 0.9336, Additional file 1: Figure S1B). Therefore, the results proved that this model was accurate enough to provide candidate promoters with specific expression efficiency for further screening. Furthermore, the relative RFP expression levels of the Constitutive Promoter Subseries I promoters were approximately 1/500 to 1/6 (without logarithmic transformation) of that of PJ23119, a commonly used strong promoter in synthetic biology [21]. Thus a promoter designed by a thermodynamic model with a predicted expression level of 7.74 might be similar to the promoter PJ23119. Based on a thermodynamic model, 61 promoters were designed with predicted expression levels that ranged from 2.00 to 8.00 and referred to as Promoter Subseries II in this article (Additional file 2: Table S3).

### The discovery of the promoter-slope parabola principle

**Fig. 2:**
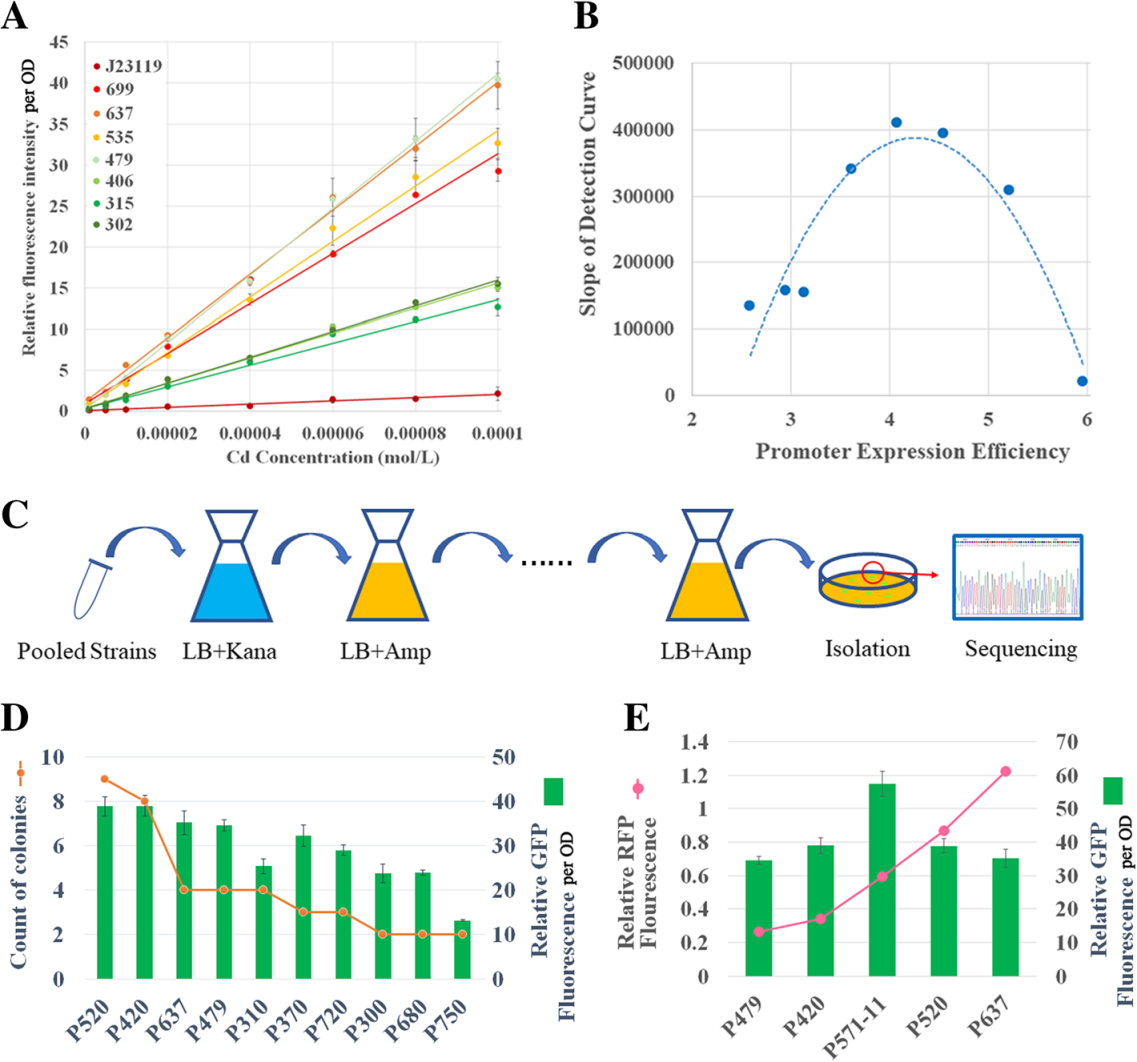
The Discovery and Verification of the Promoter-Slope Parabola Principle. **a** The performance of cadR biosensors with different constitutive promoters to initiate the expression of cadR. **b** The relationship between log values of the expression efficiency of cadR promoters (note that the x-axis represents the measured values but not the predicted values, y-axis represents the slope of fitting curve in the panel A) and the slopes of the detection curves. **c** The procedures and (**d**) results of the bottom-up pooled screening method for the selection of cadR promoters. **e** The top-down method based on the Parabola Principle for the discovery of optimum cadR promoters for highly efficient metal detection circuits

The seven promoters of the Constitutive Promoter Subseries I and PJ23119 were respectively used as Promoter X of the Cd (II) biosensor frame to form eight Cd (II) detection biosensors. Furthermore, the eGFP fluorescence intensities of these biosensors in different Cd (II) concentrations were determined and normalized to the OD600 of the culture. As shown in Fig. 2a, the relative fluorescence intensities per OD of the culture (RFI per OD) of all these biosensors indicated a positive linear relationship with increasing Cd (II) concentrations in the range of 1 μM to 100 μM. The slope of the linear detection curve could be used to access the detection sensitivity, since the larger gradient signified higher discrimination of different Cd (II) concentrations relating to fluorescence intensities. Initially, the biosensor with the strong promoter PJ23119 (named Biosensor PJ23119*-cadR*) was expected to display the best Cd (II) detection efficiency. This expectancy was due to the higher abundance of intracellular CadR levels, and therefore the higher Cd (II) ion capture capacity. However, the slope of Biosensor PJ23119*-cadR* was surprisingly the lowest among all eight Cd (II) biosensors. As overexpression of transcription factors could be toxic to bacterial cells, thus the sequence might be mutated. However, the circuits on the plasmids were further sequenced and the mutation was not observed. Additionally, the biosensors containing weak promoters for *cadR* such as P302*-cadR*, P315*-cadR*, and P406*-cadR*, displayed poor detection efficiency. The biosensors exhibiting high slope values were Biosensor P479*-cadR* and Biosensor P637*-cadR*. In both of these the expression of the *cadR* gene was initiated by a promoter with medium expression strength. Figure 2b represented the relationship between the log values of expression efficiency of the promoters for *cadR* and the slopes of the detection curves, which could be fitted by a quadratic parabolic curve (R^2^ = 0.8867). These results indicated that the detection efficiency of the Cd (II) biosensor could be improved by the optimization of intracellular CadR protein levels. A peak slope could be found at the specific intracellular sensor protein level. Furthermore, intracellular CadR concentrations that displayed levels that were either too high or too low could reduce the performance of Cd (II) biosensor. This phenomenon was referred to as the Promoter-Slope Parabola Principle and was represented by Fig. 2b. The growth rates of biosensors with different transcription factor levels were shown in Additional file 1: Figure S2. According to the results, their growth rates (OD values) showed no significant difference in cadR biosensors, thus the growth related effects on the normalized fluorescence output can be excluded.

### The verification of the promoter-slope parabola principle by bottom-up and top-down strategies

To verify the Promoter-Slope Parabola Principle in the Cd (II) biosensor, both the bottom-up and top-down experiments were conducted. During the bottom-up procedure, the pooled screening method was employed using plasmid pMetalAMP (Fig. 1c). The *egfp* gene in the Cd (II) biosensor frame was replaced by ampicillin (AMP) resistance gene *bla*. Therefore, the Cd (II) ions induced the expression of *bla*, and iterative AMP screening was utilized to locate strains with high *bla* expression levels in the Cd (II) environment (Fig. 2c). To test this screening system, the Constitutive Promoter Subseries I promoters were first pooled and transformed into pMetalAMP as promoters of SP genes. Following three iterations of the AMP screening process, the culture was diluted and plated onto Luria-Bertani (LB) agar. Ten colonies were selected for sequencing. The results showed that all these strains contained promoter P479, indicating that this system was adequate for the selection of SP promoters. To verify the Promoter-Slope Parabola Principle over a broader range, the 61 promoters from Promoter Subseries II, as well as the seven promoters from Promoter Subseries I were pooled with equal moles, and then cloned together into pMetal-AMP as promoters of SP genes, and three repetitions of the AMP screening procedure were conducted. Fifty colonies were sequenced with P520, P420, P479, P637, P310, P720, P300, P680, and P750 appearing more than once (Fig. 2d). These nine promoters were cloned into pMetalBasic and pMetalRFP, respectively. The eGFP RFI per OD of biosensors P520-*cadR* and P420-*cadR* was higher than that of P479-*cadR* and P637-*cadR* after incubation with 80 μmol/L Cd (II) (Fig. 2d). The expression levels of P520 and P420 were indicated by the relative RFP fluorescence and were located between the P479 and P637 levels (Fig. 2e), while other promoters showed respective expression levels higher than P637 or lower than P479. These results indicated that Cd (II) biosensors with a detection efficiency higher than that of Biosensor P479*-cadR* and P637*-cadR* were those whose promoter of SP presented an expression efficiency between P479 and P637, which were consistent with the Promoter-Slope Parabola Principle.

During the top-down approach, the Promoter-Slope Parabola Principle was verified by the successful prediction of its peak slope. According to the predicted parabolic curve in Fig. 2b, the peak slope was obtained when the log value of promoter expression efficiency reached 4.26, which corresponded with P571 according to the linear relationship between log value of promoter expression efficiency and model predicted expression level (Additional file 1: Figure S1A). Therefore, 12 promoters were designed using a thermodynamic model with their predicted expression levels at 5.71 and referred to as Promoter Subseries III (Additional file 2: Table S3), and were converted into the plasmid pMetalRFP. The relative RFP fluorescence of P571–11 was measured to be 0.5927 with its log value of promoter expression efficiency at 4.273, which was the closest to 4.258 of the 12 promoters in Promoter Subseries III. Furthermore, the promoter P571–11 was constructed into pMetalBasic. The Cd (II) biosensor P571–11-*cadR* showed higher eGFP RFI per OD than biosensor P479*-cadR* and P637*-cadR*, as well as P420*-cadR* and P520*-cadR* when incubated with 80 μmol/L of Cd (II) (Fig. 2e). Additionally, this result indicated that the “Parabola Principle” could be employed as a guideline for the exploration of optimum metal detection circuits.

### The parabola principle applies to biosensors based on other SPs of the MerR family

**Fig. 3:**
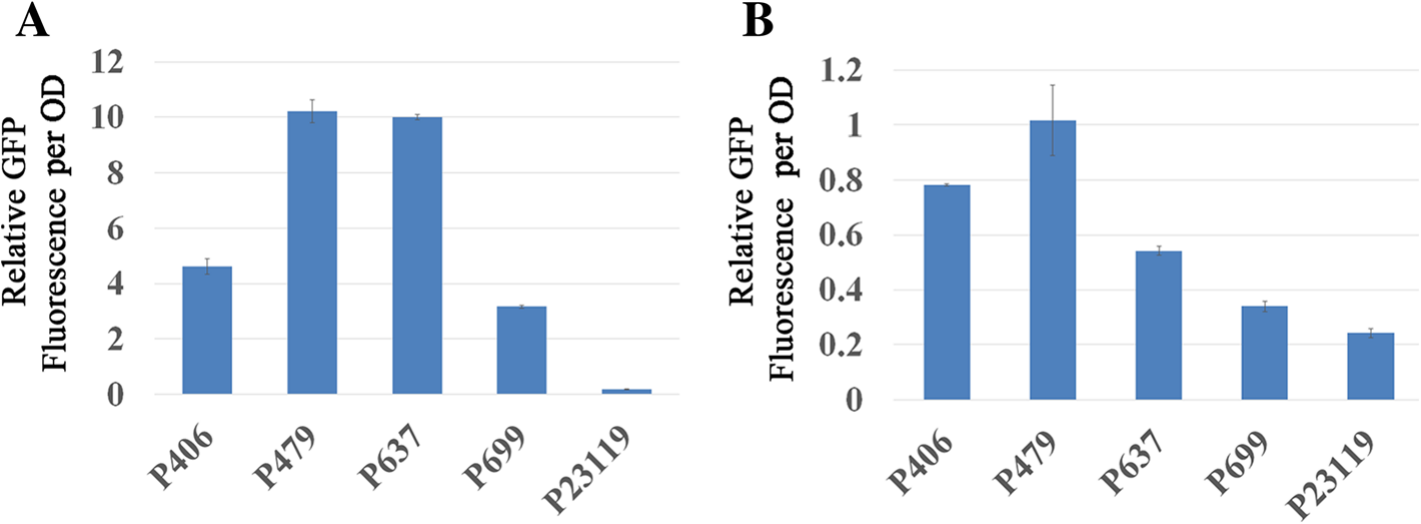
The Parabola Principle of the cueR and the pbrR based biosensors. (**a**) cueR biosensors (**b**) pbrR biosensors

To determine whether the Promoter-Slope Parabola Principle was suitable for biosensors based on other SPs in the MerR family, a series of *cueR* biosensors for Cu (I) detection and *pbrR* biosensors for Pb (II) detection were constructed. As shown in Fig. 3, the RFI per OD of both the *cueR* biosensors incubated with 10 μmol/L of Cu (I), and the *pbrR* biosensors incubated with 100 μmol/L of Pb (II) first exhibited an increase followed by a declining trend with the elevation in expression efficiency of the promoters of SPs. The relationship between the RFI per OD of the *cueR* biosensors and log values of promoter expression efficiency fitted a Parabola (R^2^ = 0.9894) without biosensors P23119-*cueR*, whose RFI per OD was approximately zero, but could not be negative to fit the Parabola. Furthermore, the RFI per OD of biosensor P406-*pbrR*, biosensor P479-*pbrR,* and biosensor P637-*pbrR,* together with their promoter expression efficiencies also fitted a Parabola. The RFI per OD of both biosensor P699-*pbrR* and biosensor P23119*-pbrR* were approximately zero. However, the ideal promoters for optimal detection efficiency in *cueR* biosensors and *pbrR* biosensors were distinctly different. The Promoter-Slope Parabola curve of the *cueR* biosensors resembled that of *cadR* biosensors, in which biosensor P479-*cueR* and biosensor P637-*cueR* displayed similar detection efficiency, and the optimum promoter was located midway between P479 and P637. However, in the Promoter-Slope Parabola of *pbrR*, the detection efficiency of biosensor P479*-pbrR* was significantly higher than that of both biosensor P637*-pbrR* and biosensor P406*-pbrR*, indicating that the optimum promoter was located close to P479. Therefore, it was speculated that the Parabola Principle applies to biosensors based on other SPs in the MerR family, while the parameters of the parabola displayed considerable diversity among disparate SPs.

### The parabola principle could be explained by the competition for SPBPs between SPs with or without ions

**Fig. 4:**
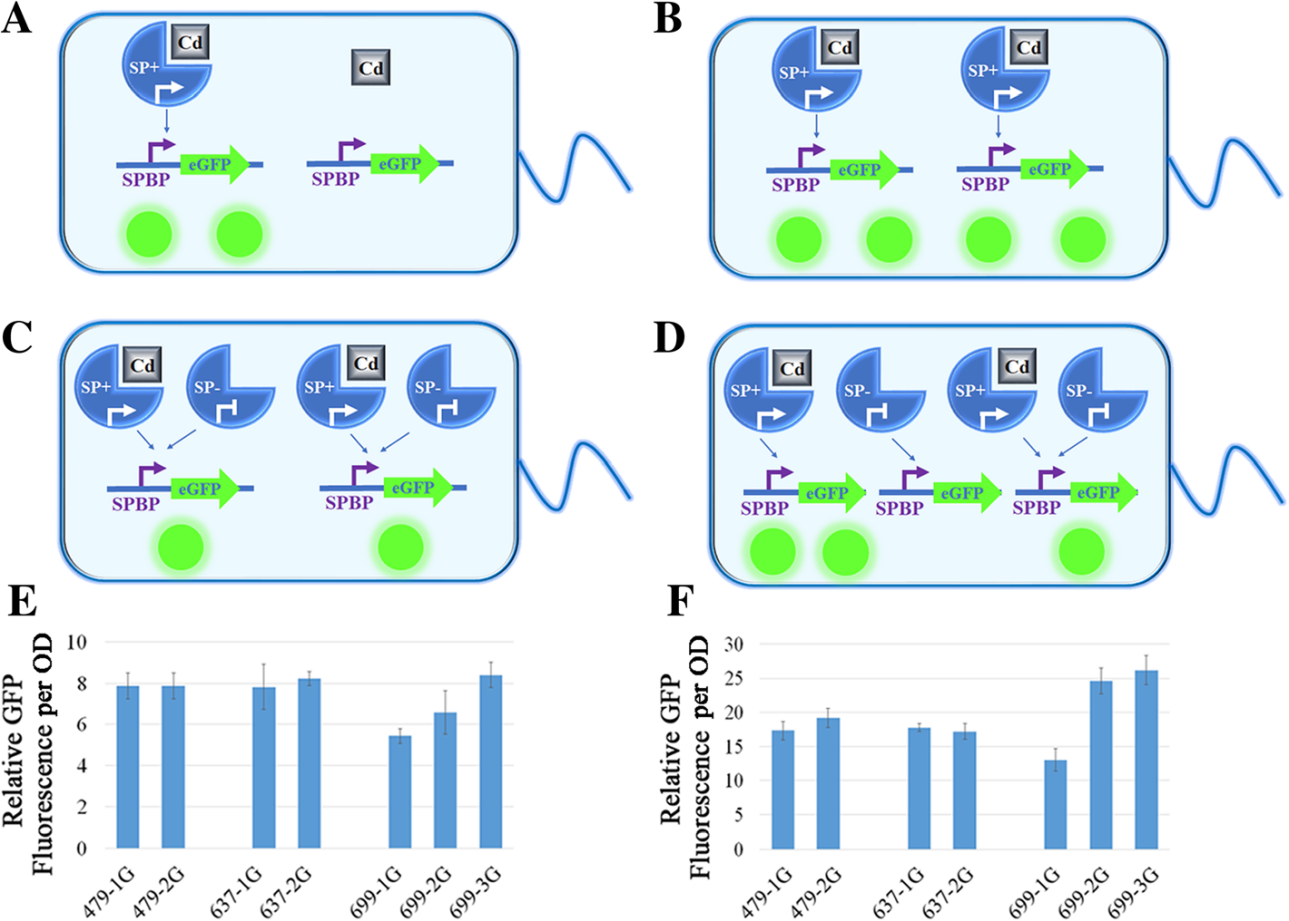
The competition hypothesis used for explaining the Parabola Principle. **a**-**c** The situations of low, optimum, and high ratios of SPs to SPBPs. **d** The situation involving the decreasing ratio of SPs to SPBPs by the introduction of additional copy numbers of SPBP-eGFP units. **e**-**f** The eGFP RFI per OD of a series of pMetalMultiGFP biosensors when incubated with 20, and 40 μmol/L of Cd (II), respectively

Furthermore, since SPs in the MerR family could bind to the corresponding SPBP in both metal-binding conformation and non-metal-binding conformation, a competition hypothesis was proposed by this study to explain the Parabola Principle. As shown in Fig. 4, changes in SP promoters resulted in intracellular molecular ratio discrepancies between SPs and SPBPs. When the expression of SPs was initiated by a weak promoter, low concentrations of intracellular SPs led to a low eGFP expression rate in the presence of metal ions meaning that the detection efficiency of heavy metal biosensors was inadequate (Fig. 4a). With the enhancement of SPs promoters, the detection efficiency of the biosensor increased until the ratio of SPs to SPBPs reached the optimum value (Fig. 4b). This optimum ratio was possibly determined by the affinity of SPs to SPBPs, which could be the reason why different SPs in the MerR family displayed varying Promoter-Slope Parabola curves. When the expression of SPs was initiated by a strong promoter, the ratio of SPs to SPBPs surpassed the optimum value. Therefore, the number of SPs exceeded the level required to bind all the intracellular metal ions. Furthermore, SPs lacking binding metal ions could create competition between SPBPs and SPs already bound to metal ions, thereby inhibiting the expression of eGFP (Fig. 4c). This competition hypothesis could explain why too high or too low intracellular SP concentrations could reduce the performance of the Cd (II) biosensor.

To preliminarily test the competition hypothesis, a series of pMetalMultiGFP plasmids (Fig. 1d) were constructed. The intention of this design was to modify the ratio of SPs to SPBPs by increasing the copy numbers of SPBP-eGFP units. If the competition hypothesis was a real occurrence, the detection efficiency of the biosensors belonging to the representations in Fig. 4a and Fig. 4b would not change by increasing the SPBP-eGFP without altering the limiting factor of SP levels. The situation in Fig. 4c depicts biosensors where the copy number of the SPBP-eGFP unit represented the limiting factor. It is possible that the introduction of more SPBP-eGFP could increase detection efficiency by relieving the competition between the SPs with or without metal binding (Fig. 4d). These results were observed when the concentrations of Cd (II) were either 20 μmol/L or 40 μmol/L as shown in Fig. 4e and Fig. 4f. With these Cd (II) ion concentrations, biosensor P479-*cadR*-2G (where 2G indicated carrying two copies SPBP-eGFP units) and biosensor P637-*cadR*-2G showed no significant increase in eGFP RFI per OD when compared to biosensor P479-*cadR* and biosensor P637-*cadR* respectively. However, biosensor P699-*cadR*-3G and biosensor P699-*cadR*-2G exhibited more eGFP than biosensor P699-*cadR*. As shown in Fig. 2b, P479 and P637 reached the approximate optimum promoter level in the Promoter-Slope Parabola curves of the SPBP-eGFP units with one copy, and P699 led to a high ratio of SPs to SPBPs, confirming that the competition hypothesis indeed occurred within a specified range of Cd (II) concentrations.

### The construction of high-performance visible qualitative hg (II) biosensors using the parabola principle

**Fig. 5:**
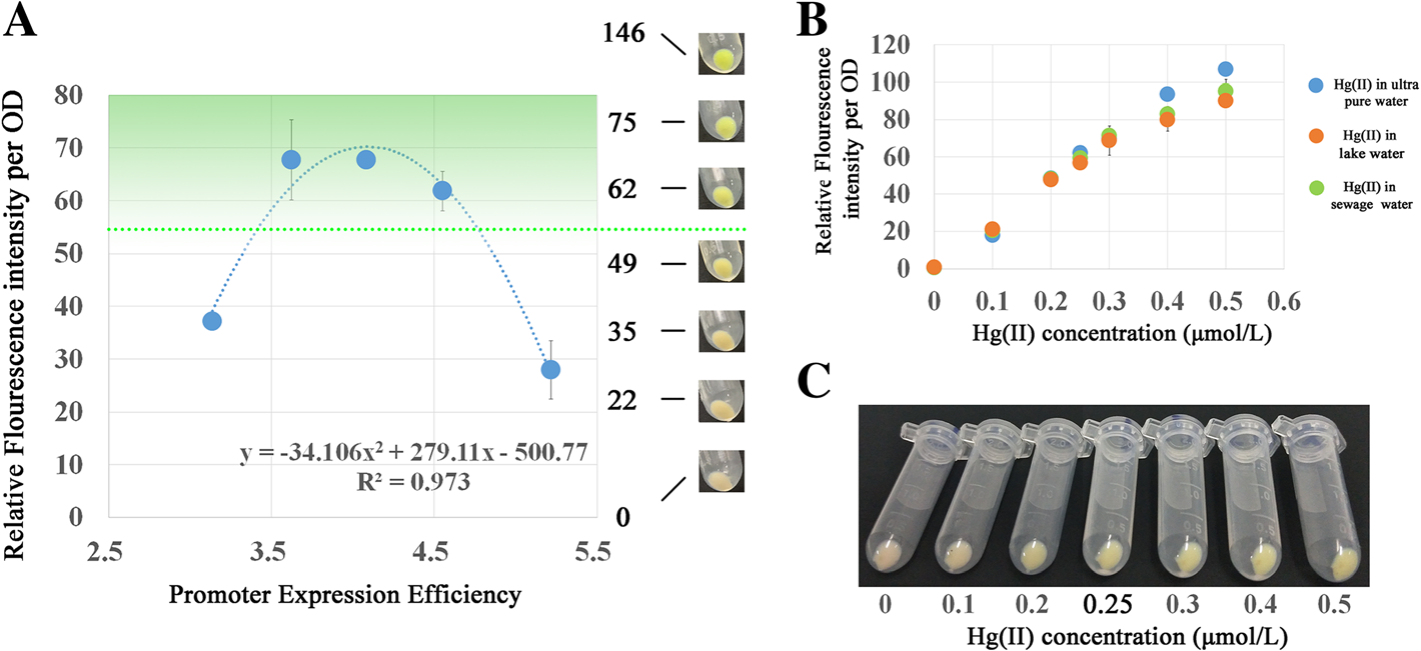
Using the Parabola Principle to design visible qualitative Hg (II) biosensors. **a** Parabola of the merR biosensors incubated with 0.25 μmol/L of Hg (II). **b** The performance of biosensor P429-merR in ultrapure water, natural lake water, and polluted water. **c** Naked eye observation of biosensor P429-merR incubated with Hg (II) solutions

To further illustrate the application of the Parabola Principle, a visible *merR* biosensor was designed for the qualitative detection of Hg (II) in natural water. In China, the limit standard for total mercury in natural water was 0.05 mg/L, which approximates to 0.25 μmol/L. Five *merR* biosensors were constructed using P406, P479, P535, P637, P699 as promoters of the *merR* genes. A volume of 4.5 mL biosensor cultures (OD600 = 0.6), 4.5 mL fresh LB broth, and 6 mL 0.25 μmol/L Hg (II) solution were mixed. Their respective fluorescence intensities were measured after three hours of incubation at 37 °C and 220 rpm. As shown in Fig. 5a, the curve of the fluorescence intensity and log values of promoter expression efficiency still fitted a parabola (R^2^ = 0.973). When the fluorescence intensity of the biosensor cultures reached approximately 55 RFI per OD, the centrifugation of the 2 mL biosensor culture (OD 600 was about 0.8 after 3 h of incubation) showed visible green coloration that was detectible by the naked eye (Fig. 5a). The distinct green line and the parabola crossed when the log values of promoter expression efficiency was at 3.423 or 4.761, which corresponded to P429 or P657 respectively according to the linear relationship between log value of promoter expression efficiency and model predicted expression level **(**Additional file 1: Figure S1A). Since a higher intracellular SP level would require more resources from the bacterial cells and result in potential mutation-selection pressure, P429 was selected as the promoter for the *merR* gene in qualitative Hg (II) detection biosensors.

For the thermodynamic model, six P429 candidates were designed referred to as Promoter Subseries IV (Additional file 2: Table S3), and they were constructed into the plasmid pMetalRFP. The relative RFP fluorescence of P429–1 was 0.0818 with its log values of promoter expression efficiency at 3.413, which was the closest to 3.423 among the Promoter Subseries IV possibilities. Using P429–1, biosensor P429-*merR* was constructed. A series of Hg (II) solutions with concentrations ranging from 0.1 to 0.6 μmol/L were prepared using ultrapure water, Hg (II)-free natural water (from an artificial lake in Beijing Olympic Forest Park), and Hg (II)-free industrial sewage water (from a rubber factory) respectively as solvents. Biosensor P429-*merR* displayed a similar detection curve in both the ultra-pure water, natural lake water solutions and industrial sewage water (Fig. 5b), which indicated biosensor P429-*merR* possessed excellent anti-interference capabilities in a natural water environment. As shown in Additional file 1: Figure S3A, at the concentration of 0.25 μmol/L, only Hg (II) could results in generaion of flourescence, biosensor cells incubated with other metals such as Cd (II), Pb (II), Fe (II), Zn (II), Cu (I), Co (I), Ni (I), or Ag (I) didn’t not show significant fluorescence signals compared with cells incubated with ultra-pure water (P > 0.05). What’s more, addition of metal ions into the Hg (II) detection system didn’t have significant influence on the generation of fluorescence signals (Additional file 1: Figure S3B, P > 0.05). These results further revealed the anti-interference capabilities of biosensor P429-merR. In the natural water solution, 0.25 μmol/L of Hg (II) resulted in 56.83 RFI per OD, and in the industrial sewage, 0.25 μmol/L of Hg (II) resulted in 59.33, which was close to the expected 55 RFI per OD. Biosensor P429-*merR* exhibited a green coloration that was visible to the naked eye when the Hg (II) concentration increased from 0.2 to 0.25 μmol/L. Therefore, this biosensor could provide a simple, fast and low-cost method to monitor Hg (II) concentrations in natural water without requiring complicated instruments and techniques. Although eGFP was not an ideal reporter for nake eyes, this example could adequately prove the feasibility of developing a metal detection biosensor with specific detection sensitivity by “Parabola Principle”.

## Conclusions

In this study, a “Parabola Principle” is proposed for the rational and purposeful design of metal detection circuits based on sensor proteins in the MerR family. The main reasoning behind the “Parabola Principle” is that the intracellular sensor protein levels can be used as a “rheostat” to adjust the metal detection slopes. When the intracellular sensor proteins reach levels that are either too high or too low, it reduces the performance of the biosensor. Furthermore, a parabola represents the fits curves between the detection slopes and the sensor protein levels. This principle has been verified by conducting both bottom-up and top-down strategy experiments, and can be explained by the competition hypothesis. Therefore, using the “Parabola Principle”, biosensors with specific detection characteristics can be designed.

## Methods

### Bacterial strain, plasmids, and oligonucleotides

*Escherichia coli* DH5α was used as receptor cells for all the plasmids in this study. Genes and oligonucleotides used in this study are listed in Additional file 2: Table S1-S3. Genes were synthesized by Genewiz Inc. (South Plainfield, USA). Oligonucleotide synthesis and plasmid sequencing were conducted by Ruibio Biotech (Beijing, China).

This research employed a thermodynamic model to design a series of constitutive promoters according to Brewster’s report [20], which gave a energy matrix of the base pairs from − 41 to − 1 upstream the transcription start sites. To generate promoters with different expression strengths, the base pairs from − 41 to − 31 and from − 12 to − 1 of the promoter were generated by random selection from A, T, C, or G, while the base pair between − 30 to − 13 of the promoter were kept as CTTTATGCTTCCGGCTCG. The RNAP binding energy of these promoters were calculated and the promoters were ranked by their RNAP binding energy to form a promoter library. Promoters with specific expression strengths were picked from the promoter library.

### Genetic circuit construction

The core circuit, composed of four elements including the *egfp* gene, SPBP, Promoter X, and the SP gene, was first synthesized and integrated with the replication origin and the kanaR from pENTR/D-TOPO (Thermo Fisher, Waltham, USA) to form pMetalBasic (Fig. 1a). Each element was flanked by restriction sites for element replacement. For the construction of the metal detection biosensors, *cadR*, *pbrR*, and *cueR* genes were respectively inserted at the SP gene site using *spe*I and *hind*III. Their respective binding promoters were inserted at the SPBP site using *bgl*II and *xho*I, while different constitutive promoters were inserted at the Promoter X site by employing *xho*I and *spe*I. For the construction of pMetal-RFP (Fig. 1b), the *rfp* gene was inserted at the SP gene site of biosensor-*cadR* using *spe*I and *hind*III. For the construction of pMetal-AMP (Fig. 1c), the *egfp* gene in biosensor-*cadR* was replaced by the *bla* gene using *kpn*I and *bgl*II. In constructing pMetal-MultiGFP, an SPBP-eGFP unit was repeatedly inserted into biosensor-*cadR* between *xba*I and *hind*III using the isocaudomer technique. LB broth, supplemented with Kanamycin (50 μg/mL), was used between genetic manipulations for overnight growth at 37 °C and 220 rpm. A heat shock procedure was employed for the transformation of *E. coli* DH5α strains.

### Biosensor preparation and fluorescence measurement

The biosensors were first activated by overnight growth in LB broth with Kanamycin. Before the bioassay, the activated biosensors were diluted into fresh LB broth containing Kanamycin and incubated at 37 °C and 220 rpm until the OD600 reached approximately 0.6. Then 5 mL biosensor cultures, 5 mL fresh LB broth with Kanamycin, and 100 μL of heavy metal solution were mixed in 50 mL flasks and incubated at 37 °C and 220 rpm for 2 h. 200 μL of cultures were selected for the measurement of OD600. Another 2 mL of the cultures were taken and centrifuged at 10000×g, and sediment was suspended again with 200 μL of 0.9% saline. The fluorescence intensity of this solution was measured using a full-wavelength spectral scanner (Thermo Fisher, Waltham, USA). For the eGFP, the excitation wavelength was 488 nm, and the emission wavelength was 507 nm, while the excitation wavelength was 587 nm, and the emission wavelength was 610 nm for the RFP. Additionally, 5 mL of the biosensor cultures, 5 mL fresh LB broth with Kanamycin, and 100 μL distilled water were mixed and incubated as the background OD and fluorescence intensity. The RFI per OD was calculated by (sample fluorescence intensity/ sample OD) – (background fluorescence intensity/ background OD).

### Bottom-up screening procedure

The promoters of the SPs were synthesized as the primer pairs. Through gradient annealing, the overlap of primer pairs formed the promoter region and the overhangs formed sticky ends consisting of xhoI and speI. Different promoters were then pooled and mixed with xhoI and speI digested plasmid PJ23119-cadR. Following the T4 ligase reaction, the resulting product was transformed into *E. coli* DH5α and cultured in LB broth with Kanamycin until the OD600 reached 0.4. Then 200 μL of the culture was selected and added to fresh LB broth with 200 μg/mL ampicillin for the first screening of the sample. When the OD600 of the cultures reached 0.6, 200 μLculture was added to fresh LB broth with the same concentration of ampicillin for the second screening process. This screening procedure was repeated several times after which 200 μL of the culture was plated onto LB agar with Kanamycin. The plasmids of the colonies were sequenced, and their Promoters of SPs were analyzed.

### Data analysis

Each procedure was repeated three times, and all values were expressed as the arithmetic mean ± standard deviation (S.D.). The data was analyzed using a one-way analysis of variance (ANOVA) followed by a t test.

## Additional files

Additional file 1: **Figure S1.** The relationship between RFP expression levels (log values). with thermodynamic model predicted expression levels (A) or with LacZ expression levels (B). **Figure S2.** The growth rates (OD values after 2 h incubation) of CadR biosensors (A) and MerR biosensors (B). No significant difference was observed. **Figure S3.** (A) Biosensor P429-merR incubated with 0.25 μmol/L of Hg (II) or other metal ions. (B) Biosensor P429-merR co-incubated with 0.25 μmol/L of Hg (II) and 0.25 μmol/L interference ions. (DOCX 544 kb)

Additional file 2: **Table S1.** Sensor Genes used in this study. **Table S2.** Sensor Protein Binding Promoters Used In This Study. **Table S3.** Constitutive Promoters Used In This Study. (DOCX 26 kb)

## Data Availability

All data generated or analysed during this study are included in this published article and its Additional files.
