# Supplementary material for: Using the promoters of MerR family proteins as “rheostats” to engineer whole-cell heavy metal biosensors with adjustable sensitivity
Source: J Biol Eng. 2019 Aug 20;13:70. doi: 10.1186/s13036-019-0202-3 (PMC6702742; doi:10.1186/s13036-019-0202-3)
Supplement: Supplementary file 1 — Figure S1. The relationship between RFP expression levels (log values). with thermodynamic model predicted expression levels (A) or with LacZ expression levels (B). Figure S2. The growth rates (OD values after 2 h incubation) of CadR biosensors (A) and MerR biosensors (B). No significant difference was observed. Figure S3. (A) Biosensor P429-merR incubated with 0.25 μmol/L of Hg (II) or other metal ions. (B) Biosensor P429-merR co-incubated with 0.25 μmol/L of Hg (II) and 0.25 μmol/L interference ions. (DOCX 544 kb) [file 13036_2019_202_MOESM1_ESM.docx]

Fig. S1 The relationship between RFP expression levels (log values). with thermodynamic model predicted expression levels (A) or with LacZ expression levels (B).


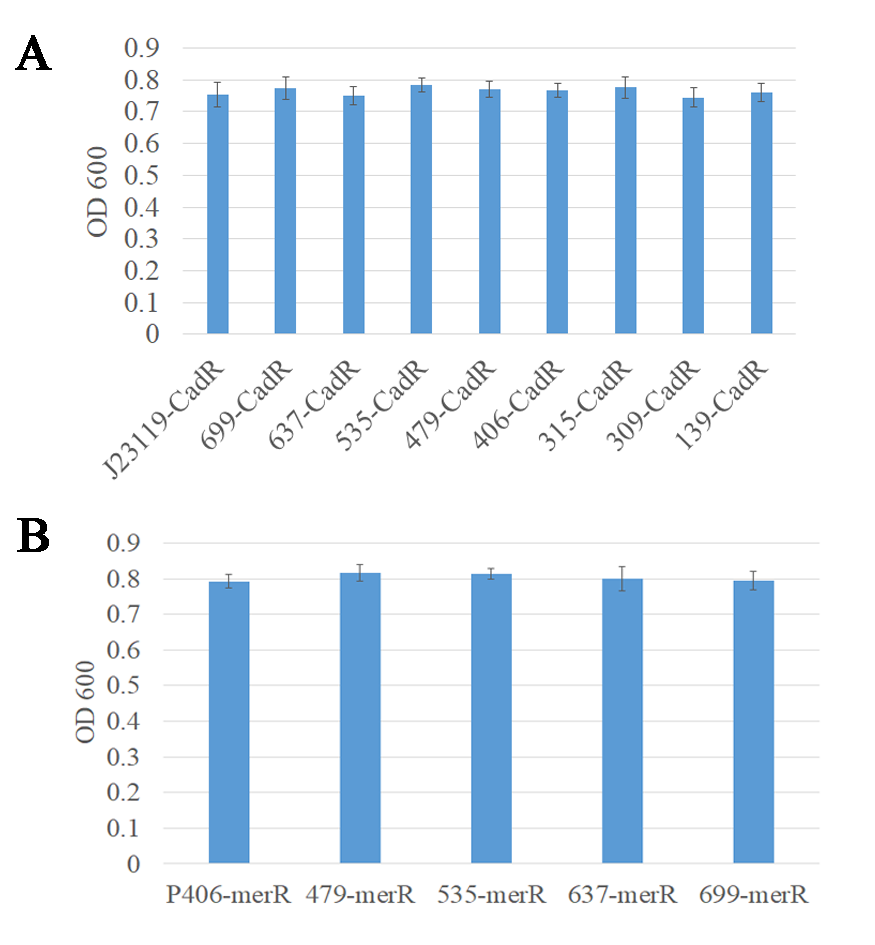


Fig. S2 the growth rates (OD values after 2 hours incubation) of CadR biosensors (A) and MerR biosensors (B). No significant difference was observed.


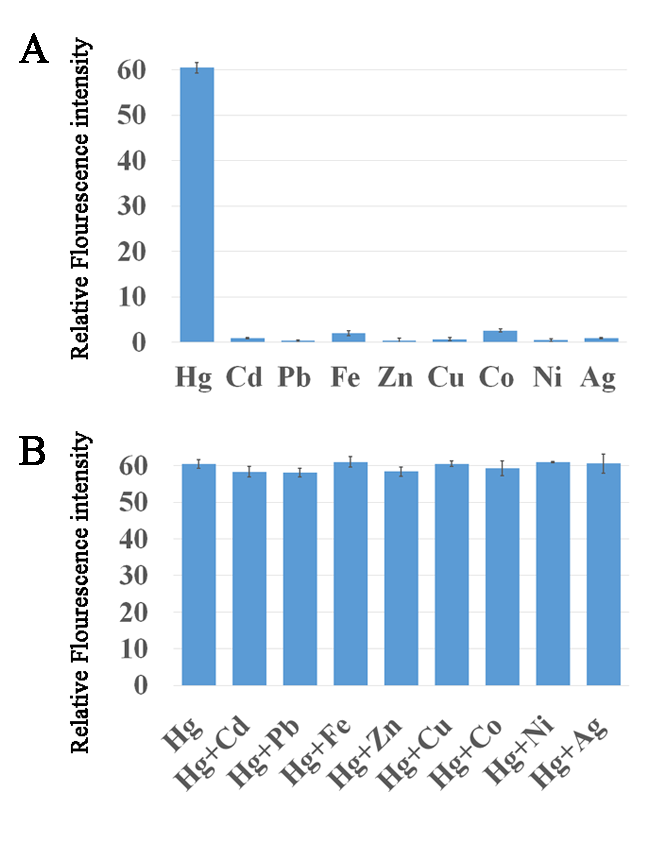


Fig. S3 (A) Biosensor P429-*merR* incubated with 0.25 μmol/L of Hg (II) or other metal ions. (B) Biosensor P429-*merR* co-incubated with 0.25 μmol/L of Hg (II) and 0.25 μmol/L interference ions.
