# Supplementary material for: Using the promoters of MerR family proteins as “rheostats” to engineer whole-cell heavy metal biosensors with adjustable sensitivity
Source: J Biol Eng. 2019 Aug 20;13:70. doi: 10.1186/s13036-019-0202-3 (PMC6702742; doi:10.1186/s13036-019-0202-3)
Supplement: Supplementary file 2 — Table S1. Sensor Genes used in this study. Table S2. Sensor Protein Binding Promoters Used In This Study. Table S3. Constitutive Promoters Used In This Study. (DOCX 26 kb) [file 13036_2019_202_MOESM2_ESM.docx]

**Supplementary Tables**

**Table S1 Sensor Genes used in this study**

| **Sensor Genes** | | **Source** |
| --- | --- | --- |
| *cadR* | ATGAAAATCGGCGAACTGGCGAAACGCACCGGTTGCCCGGTGGAAACCATCCGCTACTACGAACGCGAAGGTCTGCTGCCGGAACCAGCCCGCAGCGAAGGCAATTACCGCCAGTACACTTTAGCCCACGTTGAACGTTTGTCTTTTATCCGCCACTGCCGCTCTTTAGATATGACCCAAGAAGAAATCCGCACTTTACTGGCGCTGCGCGATCGTCCAGAAGCCGATTGCGGCACCGCCAATCGTTTAATTGATGAACATTTACACCACGTGGAAGTTCGCATCGCCGAACTGCAAGCCTTACGCGAACAGCTGCGCGATCTGGGTAGCCGCTGCACCGTTGCCGGCAATAGCCAAGCATGTGGCATTTTACGCGAACTGGAACAGCCGGCCCCACTGAGCCCAATCGCCGAAGAATGCGCCGAAGCCGGCCACATGCATGTGCCCGGTGTTCACCGCCGCCATGGCTAA | GeneBank CP020560.1  Sequence has been optimized for expression in *E. coli* |
| *cueR* | ATGAATATTAGCGATGTGGCCAAAATTACCGGTTTAACCAGCAAAGCCATCCGCTTCTATGAGGAGAAAGGTTTAGTTACCCCGCCGATGCGCAGCGAAAATGGCTATCGCACCTATACCCAGCAGCATTTAAATGAACTGACTTTACTGCGCCAAGCTCGTCAAGTTGGCTTTAATTTAGAGGAAAGCGGCGAGCTGGTGAATCTGTTCAATGATCCGCAGCGCCATAGCGCCGATGTTAAACGCCGCACTTTAGAAAAGGTTGCCGAAATCGAGCGCCACATTGAAGAACTGCAGAGCATGCGCGATCAGCTGCTGGCTTTAGCCAATGCATGTCCGGGTGATGACAGCGCAGACTGCCCGATTATTGAGAATCTGAGCGGCTGCTGCCATCATCGCGCCGGTTAA | GeneBank CP034595.1  Sequence has been optimized for expression in *E. coli* |
| *pbrR* | ATGAATATTCAGATCGGCGAGCTGGCAAAACGCACCGCCTGTCCGGTTGTGACCATTCGCTTCTACGAGCAGGAAGGCTTACTGCCGCCGCCTGGCCGTAGTCGTGGTAATTTCCGCCTGTATGGCGAAGAACACGTGGAACGCCTGCAGTTTATCCGCCATTGCCGCAGCCTGGACATGCCGCTGAGCGATGTGCGTACCCTGCTGAGCTATCGCAAACGCCCGGATCAGGATTGCGGCGAAGTGAACATGCTGCTGGATGAACATATCCGCCAGGTGGAGAGCCGTATTGGCGCCCTGCTGGAACTGAAACACCATCTGGTGGAGCTGCGTGAAGCATGTAGCGGTGCACGCCCGGCACAGAGCTGTGGTATTCTGCAGGGTCTGAGCGATTGTGTGTGCGATACCCGTGGTACCACCGCCCATCCGAGCGATTAA | GeneBank CP026545.1  Sequence has been optimized for expression in *E. coli* |
| *merR* | ATGGAAAACAATCTGGAGAACCTGACCATCGGCGTGTTTGCACGCACCGCAGGCGTGAACGTGGAAACCATCCGCTTCTATCAGCGCAAAGGCCTGCTGCCGGAACCGGATAAACCGTATGGTAGCATTCGCCGCTATGGTGAAACCGACGTGACCCGTGTGCGTTTTGTGAAAAGCGCCCAGCGTCTGGGCTTTAGCCTGGATGAAATCGCCGAACTGCTGCGTCTGGAGGATGGTACCCATTGCGAAGAAGCCAGCAGCCTGGCAGAACATAAACTGAAGGACGTGCGCGAACGTATGGCCGATCTGGCCCGCATGGAAGCCGTGCTGAGCGATCTGGTGTGCGCCTGCCATGCCCGTCGTGGTAATGTGAGCTGCCCGCTGATTGCCAGCCTGCAGGGTGGTGCAAGCTTAGCCGGTAGTGCCATGCCGTAA | GeneBank AM778842.1  Sequence has been optimized for expression in *E. coli* |

**Table S2 Sensor Protein Binding Promoters Used In This Study**

| **Sensor protein binding promoter (SPBP)** | | **Source** |
| --- | --- | --- |
| *cadR* SBPB | TTGACTCTGTAGTTGCTACAGGGTGTGCAATCGGCAAGG | **Brocklehurst et al., 2003** |
| *cueR* SPBP | TTGACCTTCCCCTTGCTGGAAGGTTTAACCTTTATCACA | **Outten et al., 2000** |
| *pbrR* SPBP | TTGACTCTATAGTAACTAGAGGGTGTTAAATCGGCAAGG | **Hobman et al., 2012** |
| *merR* SPBP | CGCTTGACTCCGTACATGAGTACGGAAGTAAGGTTACGCTAT | **Brown et al., 2003** |

**Table S3 Constitutive Promoters Used In This Study**

| **Constitutive Promoter Subseries I** | | **Source** |
| --- | --- | --- |
| P302 | CAGGCCTTAGACTTTATGCTTCCGGCTCGTATGTTGTGTGG | **Brewster et al., 2012** |
| P315 | CAGGCTTAAGACTTTATGCTTCCGGCTCGTATGTTGTGTGG | **Brewster et al., 2012** |
| P406 | TCGAGTTTACACTTTATGCTTCCGGCTCGGATAACGTGTGG | **Brewster et al., 2012** |
| P479 | TCGAGTTAAGACTTTATGCTTCCGGCTCGTATAATGTGTGG | **Brewster et al., 2012** |
| P535 | CAGGCTTTACACTTTATGCTTCCGGCTCGTATGTTGTGTGG | **Brewster et al., 2012** |
| P637 | TCGAGATTACACTTTATGCTTCCGGCTCGTATAATGTGTGG | **Brewster et al., 2012** |
| P699 | TCGAGTTTACACTTTATGCTTCCGGCTCGTATAATGTGTGG | **Brewster et al., 2012** |
| **Constitutive Promoter Subseries II** | | **Source** |
| P200 | CTATTCTTAGACTTTATGCTTCCGGCTCGTATGTTTTAGCA | This study |
| P210 | CCATTCTTAGACTTTATGCTTCCGGCTCGTATGTTGTTGGA | This study |
| P220 | CTATTCTTAGACTTTATGCTTCCGGCTCGTATGTTGTTAGT | This study |
| P230 | TCCTACTTAGACTTTATGCTTCCGGCTCGTATGTTGCACGT | This study |
| P240 | GGTATCTTAGACTTTATGCTTCCGGCTCGTATGTTTTCCGT | This study |
| P250 | TTAGACTTAGACTTTATGCTTCCGGCTCGTATGTTTTGTGC | This study |
| P260 | CGGATCTTAGACTTTATGCTTCCGGCTCGTATGTTCGTAAT | This study |
| P270 | CTACTCTTAGACTTTATGCTTCCGGCTCGTATGTTAACTAA | This study |
| P280 | GCACTCTCAGACTTTATGCTTCCGGCTCGTATAATACTTGG | This study |
| P290 | CGTGTCTCAGACTTTATGCTTCCGGCTCGTATAATGCGCGT | This study |
| P300 | GGGCGCTTAGACTTTATGCTTCCGGCTCGTATGTTACTCCA | This study |
| P310 | TACCTCTCAGACTTTATGCTTCCGGCTCGTATAATGCTAAG | This study |
| P320 | ACGCCCTCAGACTTTATGCTTCCGGCTCGTATAATGCGACA | This study |
| P330 | CCCGACTCAGACTTTATGCTTCCGGCTCGTATAATCGGCAC | This study |
| P340 | CATCACTTAGACTTTATGCTTCCGGCTCGTATGTTATGCAT | This study |
| P350 | GGTTACTCAGACTTTATGCTTCCGGCTCGTATAATTGAACC | This study |
| P360 | ACTAACTTAGACTTTATGCTTCCGGCTCGTATGTTATTTAA | This study |
| P370 | CGACCCTCAGACTTTATGCTTCCGGCTCGTATAATGATACT | This study |
| P380 | CGTCGCTCAGACTTTATGCTTCCGGCTCGTATAATCCCTAA | This study |
| P390 | AGCCCCTCAGACTTTATGCTTCCGGCTCGTATAATGTAGGG | This study |
| P400 | TCCTAATTACACTTTATGCTTCCGGCTCGTATGTTCCGAAG | This study |
| P410 | TAGGTATTACACTTTATGCTTCCGGCTCGTATGTTGTTCGC | This study |
| P420 | AGTTAATTACACTTTATGCTTCCGGCTCGTATGTTGCGCGA | This study |
| P430 | GGGGGCTCAGACTTTATGCTTCCGGCTCGTATAATTTCGAC | This study |
| P440 | CTTCAATTACACTTTATGCTTCCGGCTCGTATGTTTTAATA | This study |
| P450 | TGCAAATTACACTTTATGCTTCCGGCTCGTATGTTCAAAGC | This study |
| P460 | AAACTATTACACTTTATGCTTCCGGCTCGTATGTTAAGGAG | This study |
| P470 | CAGGCATTACACTTTATGCTTCCGGCTCGTATGTTACCTTC | This study |
| P480 | CAACGATTACACTTTATGCTTCCGGCTCGTATGTTTTAAGT | This study |
| P490 | GTTAGATTACACTTTATGCTTCCGGCTCGTATGTTGTCCTA | This study |
| P500 | CTCTTATTACACTTTATGCTTCCGGCTCGTATAATCAGCCA | This study |
| P510 | GCATGATTACACTTTATGCTTCCGGCTCGTATGTTGTACAA | This study |
| P520 | TATCGATTACACTTTATGCTTCCGGCTCGTATGTTCAAGAC | This study |
| P530 | AAATTATTACACTTTATGCTTCCGGCTCGTATAATCGGATG | This study |
| P540 | GACGTATTACACTTTATGCTTCCGGCTCGTATAATCGTACG | This study |
| P550 | GTCCTATTACACTTTATGCTTCCGGCTCGTATAATGCGAAG | This study |
| P560 | GCGCAATTACACTTTATGCTTCCGGCTCGTATAATTACCTG | This study |
| P570 | CTGTTATTACACTTTATGCTTCCGGCTCGTATAATATTCGT | This study |
| P580 | ACACTTTTACACTTTATGCTTCCGGCTCGTATAATGCGATA | This study |
| P590 | TTTGAATTACACTTTATGCTTCCGGCTCGTATAATACCCTG | This study |
| P600 | TTGCGATTACACTTTATGCTTCCGGCTCGTATAATCACTCA | This study |
| P610 | CTGGCATTACACTTTATGCTTCCGGCTCGTATAATTATATC | This study |
| P620 | CGTGGATTACACTTTATGCTTCCGGCTCGTATAATCCGATT | This study |
| P630 | CTGCTTTGACACTTTATGCTTCCGGCTCGTATAATTCGCCC | This study |
| P640 | CCGCGTTTACACTTTATGCTTCCGGCTCGTATAATTCGTGT | This study |
| P650 | TGATGTTTACACTTTATGCTTCCGGCTCGTATAATTCGTCA | This study |
| P660 | TAGTCATTACACTTTATGCTTCCGGCTCGTATAATAGATCT | This study |
| P670 | GTGTATTTACACTTTATGCTTCCGGCTCGTATAATCGCTAT | This study |
| P680 | CGCGAATTACACTTTATGCTTCCGGCTCGTATAATAACAAA | This study |
| P690 | TAAACTTTACACTTTATGCTTCCGGCTCGTATAATTTTGTA | This study |
| P700 | TCTTCTTTACACTTTATGCTTCCGGCTCGTATAATATGAGG | This study |
| P710 | TGATTTTGACACTTTATGCTTCCGGCTCGTATAATGTGCCT | This study |
| P720 | ACACGTTTACACTTTATGCTTCCGGCTCGTATAATAGTATT | This study |
| P730 | TAGCGTTTACACTTTATGCTTCCGGCTCGTATAATAGAACC | This study |
| P740 | GGTTATTGACACTTTATGCTTCCGGCTCGTATAATTGCCGC | This study |
| P750 | CTCGCTTTACACTTTATGCTTCCGGCTCGTATAATATTAAC | This study |
| P760 | AACGATTGACACTTTATGCTTCCGGCTCGTATAATCTTCGT | This study |
| P770 | CAAGTTTGACACTTTATGCTTCCGGCTCGTATAATGTCGAT | This study |
| P780 | GCAAATTGACACTTTATGCTTCCGGCTCGTATAATGGTCAA | This study |
| P790 | TGATCTTGACACTTTATGCTTCCGGCTCGTATAATCGCGAT | This study |
| P800 | CGTGGTTGACACTTTATGCTTCCGGCTCGTATAATTTTCTG | This study |
| **Constitutive Promoter Subseries III** | |  |
| \| P571-1 \| TCAATTTTACACTTTATGCTTCCGGCTCGTATAATCACCGG \| This study \| \| --- \| --- \| --- \| | | This study |
| \| P571-2 \| TCTCTATTACACTTTATGCTTCCGGCTCGTATAATTGTAAA \| This study \| \| --- \| --- \| --- \| | | This study |
| \| P571-3 \| CTGCTATTACACTTTATGCTTCCGGCTCGTATAATAAAGCG \| This study \| \| --- \| --- \| --- \| | | This study |
| \| P571-4 \| AAAGTTTTACACTTTATGCTTCCGGCTCGAATAATGGAGCA \| This study \| \| --- \| --- \| --- \| | | This study |
| \| P571-5 \| TCGAATTTACACTTTATGCTTCCGGCTCGAATAATTTCGCA \| This study \| \| --- \| --- \| --- \| | | This study |
| \| P571-6 \| CCCGCTTTACCCTTTATGCTTCCGGCTCGTATAATGTCACG \| This study \| \| --- \| --- \| --- \| | | This study |
| \| P571-7 \| TTCTGTTTACCCTTTATGCTTCCGGCTCGTATAATCAACGA \| This study \| \| --- \| --- \| --- \| | | This study |
| \| P571-8 \| TGCAATTTACACTTTATGCTTCCGGCTCGGATAATCATTAT \| This study \| \| --- \| --- \| --- \| | | This study |
| \| P571-9 \| GGTTATTTACACTTTATGCTTCCGGCTCGGATAATATACGG \| This study \| \| --- \| --- \| --- \| | | This study |
| \| P571-10 \| AGGGGTTTACACTTTATGCTTCCGGCTCGTATGTTCGACAG \| This study \| \| --- \| --- \| --- \| | | This study |
| \| P571-11 \| AGTAGTTTACACTTTATGCTTCCGGCTCGTATGTTTATATA \| This study \| \| --- \| --- \| --- \| | | This study |
| \| P571-12 \| GCTAGTTAAGACTTTATGCTTCCGGCTCGTATAATAAATAG \| This study \| \| --- \| --- \| --- \| | | This study |
| **Constitutive Promoter Subseries IV** | |  |
| \| P429-1 \| TGTTTATTACACTTTATGCTTCCGGCTCGTATGTTGTTGGT \| This study \| \| --- \| --- \| --- \| | | This study |
| \| P429-2 \| GTTGAATTACACTTTATGCTTCCGGCTCGTATGTTTGGAGA \| This study \| \| --- \| --- \| --- \| | | This study |
| \| P429-3 \| ACATGATTACACTTTATGCTTCCGGCTCGTATGTTTACCGG \| This study \| \| --- \| --- \| --- \| | | This study |
| \| P429-4 \| CGGCTATTACACTTTATGCTTCCGGCTCGTATGTTATCTGC \| This study \| \| --- \| --- \| --- \| | | This study |
| \| P429-5 \| ATATTATTACACTTTATGCTTCCGGCTCGTATGTTGACTAA \| This study \| \| --- \| --- \| --- \| | | This study |
| \| P429-6 \| CCCTGCTCAGACTTTATGCTTCCGGCTCGTATAATATTAAG \| This study \| \| --- \| --- \| --- \| | | This study |
| **Other Constitutive Promoter** | |  |
| PJ23119 | TTGACAGCTAGCTCAGTCCTAGGTATAATGCTAGG | **Lucks et al., 2012** |

**Reference**

Brocklehurst K. R., Megit S. J., and Morby A. P. (2003) Characterisation of CadR from Pseudomonas aeruginosa : a Cd(II)-responsive MerR homologue. *Biochem. Bioph. Res. Co. 308(2)*, 234-239.

Hobman J. L., Julian D. J., and Brown N. L. (2012) Cysteine coordination of Pb(II) is involved in the PbrR-dependent activation of the lead-resistance promoter, PpbrA, from Cupriavidus metallidurans CH34. *BMC microbiol. 12(1)*, 109.

Lucks J. B., Qi L., Mutalik V. K., Wang D., and Arkin A. P. (2011) Versatile RNA-sensing transcriptional regulators for engineering genetic networks. *P. Natl. Acad. Sci. USA.* *108(21)*, 8617-8622.

Outten F. W., Outten C. E., Hale J., and O’Halloran T. V. (2000) Transcriptional activation of an Escherichia coli copper efflux regulon by the chromosomal MerR homologue, cueR. *J. Biol. Chem. 275(40)*, 31024-31029.
